# Experts’ opinion about the pediatric secondary headaches diagnostic criteria of the ICHD-3 beta

**DOI:** 10.1186/s10194-017-0819-x

**Published:** 2017-11-29

**Authors:** Aynur Özge, Ishaq Abu-Arafeh, Amy A. Gelfand, Peter James Goadsby, Jean Christophe Cuvellier, Massimiliano Valeriani, Alexey Sergeev, Karen Barlow, Derya Uludüz, Osman Özgür Yalın, Noemi Faedda, Richard B. Lipton, Alan Rapoport, Vincenzo Guidetti

**Affiliations:** 1 Department of Neurology, Mersin University Medical Faculty, Mersin, Turkey; 2 Royal Hospital for Sick Children, Glasgow, G3 8SJ UK; 3 UCSF Headache Center and UCSF Benioff Children’s Hospital Pediatric Brain Center 2330 Post St, 6th Floor, Campus Box 1675, San Francisco, CA 94115 USA; 4 NIHR-Wellcome Trust King’s Clinical Research Facility, King’s College London, London, UK; 5 Division of Paediatric Neurology, Department of Paediatrics, Lille Faculty of Medicine and Children’s Hospital, Lille, France; 6 Division of Neurology, Ospedale Pediatrico Bambino Gesù, Piazza Sant’Onofrio 4, Rome, Italy; 7 Center for Sensory-Motor Interaction Aalborg University, Aalborg, Denmark; 8 Department of Neurology and Clinical Neurophysiology, University Headache Clinic, Moscow State Medical University, Moscow, Russia; 9 Faculty of Medicine, University of Calgary, Alberta Children’s Hospital, C4-335, 2888 Shaganappi Trail NW, Calgary, AB T3B 6A8 Canada; 10 Cerrahpaşa Medical Faculty, Deaprtment of Neurology, İstanbul University, Kocamustafapaşa, İstanbul, Turkey; 11 İstanbul Research and Education Hospital, Kocamustafapaşa, İstanbul, Turkey; 12 Phd program in Behavioural Neuroscience, Department of Paediatrics and Child and Adolescent Neuropsychiatry, Sapienza University of Rome, Rome, Italy; 13 Department of Psychiatry and Behavioral Sciences, Department of Epidemiology & Population Health, Montefiore Headache Center, Albert Einstein College of Medicine, Bronx, NY USA; 14 The David Geffen School of Medicine at UCLA, Los Angeles, CA USA; 15 Department of Pediatrics and Child and Adolescent Neuropsychiatry, Sapienza University, Rome, Italy

**Keywords:** Headache, Classification, Childhood headache, Adolescent headache, Primary headache disorders, Migraine, Tension-type headache, Cluster headache

## Abstract

**Background:**

The 2013 International Classification of Headache Disorders-3 was published in a beta version to allow clinicians to confirm the validity of the criteria or suggest improvements based on field studies. The aim of this work was to review the Secondary Headache Disorders and Cranial Neuralgias and Other Headache Disorders sections of ICHD-3 beta data on children and adolescents (age 0–18 years) and to suggest changes, additions, and amendments.

**Methods:**

Several experts in childhood headache across the world applied different aspects of ICHD-3 beta in their normal clinical practice. Based on their personal experience and the available literature on pediatric headache, they made observations and proposed suggestions for the mentioned headache disorders on children and adolescents.

**Results:**

Some headache disorders in children have specific features, which are different from adults that should be acknowledged and considered. Some features in children were found to be age-dependent: clinical characteristics, risks factors and etiologies have a strong bio psychosocial basis in children and adolescents making primary headache disorders in children distinct from those in adults.

**Conclusions:**

Several recommendations are presented in order to make ICHD-3 more appropriate for use in children.

## Background

Headache is a frequent cause of pain and significant disability in children and adolescents. Its varying presentations, etiologies, triggers and methods of management can pose diagnostic and therapeutic dilemmas. Secondary headache disorders in childhood are different from the manifestations in adults, and the cause for this difference is unknown. It could be the result of the differences in degree of brain maturation comprising myelination, new synapse formation and synaptic reorganization [1–3]. There is little data about the critics of secondary headache disorders in children and adolescents in the basis of causes and cerebral maturation.

Owing to the high prevalence of childhood headache and the absence of specific objective diagnostic criteria for children, accurate clinical diagnostic criteria are needed.

## Methods

The researchers of this article consist of authors who have written at least 3 prestigious published papers about headache in children and adolescents, and are also members of the International Headache Society Pediatric Special Interest Group. Subgroups of ICHD-3 beta have been distributed among the researchers provided that each individual have at least one published article related to their assigned subgroup. Some members of this Consensus were assigned to design and review the fields of the research. All researchers analyzed the ICHD-3 beta criteria and commented on it based on the literature, providing supporting articles. The primary source of literature was Pubmed, and the paper also benefited from other widely used search engines, such as Google Scholar, and reference lists from single articles, reviews and editorials. The draft was later presented to all of the researchers for them to include their personal clinical practices and suggestions on modifying the ICHD-3 beta criteria for all clinical practitioners to be able to better diagnosis. The final draft of the Consensus article has been submitted after multiple revisions.

### Headache attributed to trauma or injury to the head and/or neck

#### Comments

The main presenting symptoms of mild traumatic head injury are headache, fatigue, and dizziness, and difficulty/slow thinking. Sleep disturbances, frustration, forgetfulness, and fatigue were most likely to first develop during follow-up. Irritability and sleep disturbances last longest (16 days), followed by frustration and poor concentration (14 days); nausea, depression, dizziness, and double vision tended to abate quickly. It is known that at 1 month after injury, nearly 25% of children still had headache, 20% still complained of fatigue, and nearly 20% were still having difficulty thinking [4–8]. These symptoms are not different from post-traumatic symptoms reported in adult patients [9].

#### Recommendations

These adult diagnostic criteria are very broad and should be emphasized in depth in children and adolescent. These criteria only help defining the relation to the trauma with its subtypes and the grading of its severity (moderate to severe or mild), however the criteria do not help showing accompanied clinical fenotypes for adolescent and pediatric patients. A modified Glasgow coma scale should be used for young children in which scoring should be different for children in order to better reflect developmental characteristics [10].

### Headache attributed to cranial or cervical vascular disorder

#### Comments

All causes of vascular headache can be seen in childhood age with some phenotypic changes. The manifestations of the syndrome of cerebral autosomal-dominant arteriopathy with subcortical infarcts and leukoencephalopathy (CADASIL) begin in childhood up to adulthood (8–60 years) patients with headache, or with a family history of early stroke or dementia or mood changes [11–15]. Since few pediatric patients have been reported those with presenting symptoms of headache had migraine with or without aura or atypical migraine. In a large study consisting of 204 pediatric patients with Moyamoya disease suffered headaches with following characteristics: nausea, vomiting, and abdominal pain. Headache could be localized or unlocalized (one parietal, and two temporal). In four patients, headache developed during hyperventilatory conditions such as exercise, crying, or taking hot food. In three of them, a TIA and headache occurred simultaneously on hyperventilation [16]. In patients younger than 18 years, the clinical manifestations of neuro-Behçet’s disease are caused mainly by cerebral sinus venous thrombosis or idiopathic intracranial hypertension and symptoms are related to this entity or due to the location of the parenchymal brain involvement [17, 18].

Intracranial hemorrhage and arterial ischemic stroke prevalence is 74% and 21% in pediatric population. However, there are no specific remarks have been described in headache disorders related to stroke-like episodes in pediatric population [19]. Trauma related cervical or carotid artery dissections are not uncommon during childhood and adolescents. Carotid or vertebral dissection is generally associated with constant unilateral pain (ipsilateral to the dissection), although throbbing, thunderclap, and gradually worsening headaches have been reported as well [20]. Warning signs of headache due to cranial or vascular disorder are as follows; first or worst headache, especially if sudden in onset; headache after effort; onset before 10 years of age; worsening during the observation period; accompanied by vomiting; presence of focal signs and papilledema; and positive family history of stroke or dementia [21].

Headache prevalence in pediatric patients with arteriovenous fistula (DAVF) is very uncommon (1 of 58 described cases) with a very young age at men presentation less than 5 years. The limited verbal expression in this age is limited and tinnitus can’t be described and we suggest changing the criteria (see Headache attributed to Dural Arteriovenous fistula) [21]. Nine cases have been reported in pediatric patients with a sparse data referring headache characteristics except thunderclap headache [22].

### Recommendations

#### Headache attributed to Dural Arteriovenous fistula

3. at least one of the following:

- headache is accompanied by tinnitus (Children beavior is more important than words, such as covering their ears)
- headache is accompanied by ophthalmoplegia
- headache is both progressive and worse in the morning and/or during coughing and/or bending over

#### Headache attributed to genetic vasculopathy

We suggest the criteria to be changed as follows:

1. Migraine like headache attacks
2. Presentation as stroke-like episodes
3. Deterioration in cognitive skills or behavior changes

### Headache attributed to non-vascualar intracranial disorder

#### Headache attributed to idiopathic intracranial hypertension (IIH)

The course of pediatric IIH varies, and a child may vary with present hours to several years after symptoms begin. While headache, nausea, and vomiting are known classic but nonspecific symptoms, patients may complain of blurred vision, diplopia, and stiff neck as well. At presentation, visual acuity loss is reported in 6%–20% of pediatric cases, although visual field loss occurs in up to 91% of cases with a careful anamnesis. Mentioned diagnostic criteria of IIH are far from diagnostic level of children and adolescents [23].

### Recommendations

Proposed criteria for pediatric IIH diagnosis are:

1. Prepubertal presentation.
2. If symptoms or signs present, they may only reflect those of generalized intracranial hypertension of papilledema with normal mental status.
3. Documented elevated intracranial pressure (**≥**250 mm CSF in adults and **≥**280 mm CSF in children, 250 mm CSF if the child is not sedated and not obese) in a properly performed lumbar puncture in the lateral decubitus position.

Neonates: ≥76 mm H_2_0.

Age < 8 years with papilledema: ≥180 mm H_2_0.

Age ≥ 8 years or < 8 years without papilledema: >250 mm H_2_0.

(4) Normal CSF composition except in neonates who may have up to 32 WBC/mm3 and protein as high as 150 mg/dL.
(5) No evidence of hydrocephalus, mass, structural, or vascular lesion on MRI, with and without contrast, and MR venography. Narrowing of the transverse sinuses is allowed.
(6) Cranial nerve palsies allowed if they are of no other identifiable etiology and improve with reduction in cerebrospinal fluid pressure or resolution of other signs and symptoms of intracranial hypertension.
(7) No other identified cause of intracranial hypertension. Children should have signs or symptoms consistent with elevated intracranial pressure not attributed to other causes [23].

#### Headache attributed to spontaneous intracranial hypotension

Spontaneous intracranial hypotension (SIH) is a condition in which a patient develops postural headaches because of a leak of the cerebrospinal fluid (CSF) in the dural membrane. The principal presenting symptom of SIH is headache. The classic definition is orthostatic headache, where severe headache attacks occur when the patient gets to an upright position, which is relieved upon lying flat. Among the features other than headache, posterior neck pain or stiffness, nausea, and vomiting are the most common, being reported by approximately 50% of patients, and suggest meningeal irritation, particularly when photophobia or phonophobia is also present. Examinations of children can reveal neck stiffness, horizontal diplopia, facial weakness, vestibulocochlear nerve abnormalities (decreased speech discrimination, disturbed diaphosone tests, disturbed posturography etc), radicular features (pain, dermatomal hypoesthesia, radicular weakness, etc), cerebellar ataxia, and encephalopathy. Children show these values approximately 20 to 30 mmH_2_O higher than adults. However, lumbar puncture is not recommended for confirmation in all cases because of the possibility of worsening of the patients’ symptoms by further reducing CSF volume [23].

### Recommendation

The following diagnostic criteria should be added to the text. “Children show initial pressure values approximately 20 to 30 mmH_2_O higher than adults in lumbar puncture procedures.”

### Syndrome of transient headache and neurological deficits with cerebrospinal fluid Lymphocytosis (HaNDL)

Review of the literature identifies 14 cases of HaNDL in the pediatric population. This syndrome may mimic much more common conditions such as complicated or hemiplegic migraine, aseptic meningitis, meningoencephalitis, or stroke. This clinical syndrome is under recognized and underreported. HaNDL remains a diagnosis of exclusion especially in children and adolescents [24]. We don’t have any recommendation for this title.

### Headache attributed to intracranial neoplasm

#### Comments

Brain tumors are rare in children, with an incidence of 5 per 100,000 in the range of from 0 to 19 years of age group. In infants, brain tumors present with fewer dramatic symptoms due to the accommodation of the skull bones when the fontanels and sutures are still open. Both the presence of a brain tumor and its management with radiation increase the risk of additional neurovascular events compared to the general pediatric population (548 per 100**,**000 vs 2**–**8 per 100**,**000) [25–27].

#### Recommendations

The following changes might be added in the diagnostic criteria for section 3 of ICHD-3 beta (Headache attributed to intracranial neoplasm) [25–27].

3. Headache has at least one of the following characteristics

- progressive worse in the morning or after daytime napping or awakes child from sleep
- projectile vomiting
- alterations in consciousness
- seizures
- starts after physical effort or worsens after the Valsalva Maneuver
- associated with endocrine alterations
- showing visual disturbance unrelated to the migraine
- persistent localized pain

### Headache attributed to epileptic seizures

Since these criteria are general and rely on cause and effect we suggest accepting the adult criteria as well [28]. “Hemicrania epileptic” is restrictive diagnosis in children and adolescents [29]. This condition is extremely rare, and when it does appear, it is unlikely to meet all proposed diagnostic criteria. Hemicrania epileptica is not listed among the nosological or terminological recommendations of the ILAE Commission on Classification and Terminology [30]. In an introductory article, Isler et al. studied 91 patients with drug-resistant epilepsy; of the total, 18 presented hemicranial headache with migraine-like characteristics lasting seconds to minutes at the time of onset of epileptic activity, which was partial seizure in all patients. In rare cases, ictal headache lasted for hours but always need EEG corroborative evidence [31].

We need more data about children and adolescents for reaching the best point.

### Headache attributed to Chiari malformation type I

#### Comments

More typically, CM-1 presents in young adults with neurological symptoms including a headache, cervical pain, cranial nerve palsies, neurosensory deficit, and ataxia. Headache is the most common presentation symptom of Chiari malformation commonly provokes by Valsalva maneuver. Most cases describe headaches similar to primary headache disorders like migraine or tension-type headaches. Only 6% of patients reported of occipital headache attacks [32–34].

#### Recommendations

We suggest that “occipital location of headache attacks is not a rule” in children and adolescents.

### Headache attributed to substance or withdrawal

There is almost no specific report has been published regarding the relation between substance abuse and headache disorders in children and adolescents. Illicit drug use may be complicated by reversible cerebral vasoconstriction syndrome manifesting as thunderclap headache [35, 36]. Alcohol induced headache reported to have migraine like headache tension cluster headache and triggers of less frequent types of primary headache such as familial hemiplegic migraine, hemicrania continua, and paroxysmal hemicrania with no specific reports specific reports in children and adolescents [36].

#### Medication overuse headache

Overuse of nonspecific analgesics is most common in adults overall and this appears to be the case in children as well. In a small study of forty-two pediatric chronic daily headache patients with medication overuse more than half were overusing non-steroidal antiinflammatory drugs (NSAIDs), about a quarter acetaminophen, and only 12% were overusing prescription medications. More data are needed to clarify which types of medications children and adolescents are overusing [37, 38].

However, since the generality of these adult criteria (Medication overuse headache) we suggest accepting these criteria up to detailed description in pediatric patients.

### Headache attributed to infection

#### Headache attributed to intracranial infection

Adult criteria are general and related to cause and effect can be used for pediatric patients.

#### Headache attributed to bacterial meningitis or meningoencephalitis

It is known that nearly 24.9% of the patients exhibited signs of meningeal irritation in children especially under 3 years of age. The prevalence of all other symptoms and signs were less than 20% [39]. On the other side, bacterial meningitis is present in 30% of children with signs of meningeal irritation. Presence of meningeal irritation assessed by the pediatrician is related to bacterial meningitis in 39%. The meningoencephalitis seldom affected children absent fever and neck stiffness. The etiology was known in only22.4% of the cases, entero viruses being the most frequent causative agent in young children [40].

Criterion-A- 4 should be changed for children as follows:

- holocranial
- located in the nuchal area and associated with neck stiffness.

These symptoms are missing in some cases of pediatric intracranial infection [41]. Some cases of children and infants, immunosuppressed patients present meningoencephalitis absent neck stiffness or any other signs of meningeal irritation.

We suggest to add the following item on the ICHD-3B criteria for the diagnosis of Headache attributed to bacterial meningitis or meningoencephalitis; some cases of meningoencephalitis could be presented without meningeal irritation signs.

### ICHD-3B Headache attributed to brain abscess

#### Comments

Fever and headache are the most common presenting symptoms of brain abscess in children and adolescents*.* The main predisposing factor is cyanotic congenital heart disease, sinusitis, otitis, or dental infections. Because of this, the location of abscess can often reveal the primary infection site. Presentations of brain abscesses are relatively nonspecific and occur due to focal mass expansion, intracranial hypertension, or diffuse destruction. Headache, fever, altered level of consciousness, nausea, vomiting, and focal neurologic deficits are common symptoms. Importantly, seizures occur in 30–50% of patients. Children and adolescents with intravenous or nasal substance users have an increased risk for brain abscess in case of patent foramen ovale [42, 43].

### Comment regard the sections "Headache attributed to brain abscess, Headache attributed to subdural, empyema, Headache attributed to systemic infection" of ICHD-3B

Since the general description of the symptoms and the relations to abscess, or subdural empyema or systemic bacterial infection systemic viral infection appearance or recovery adult criteria may be used for pediatric patients.

### Recommendations

Children and adolescents with intravenous or nasal substance users have an increased risk for brain abscess in case of patent foramen ovale.

### Headache attributed to disorders of homeostasis

#### Comments

These criteria have a good generalizability, since no specific description for pediatric headaches is given.

#### Hypoxia and/or Hypercapnia

### High-altitude headache

#### Comments

Pediatric studies on high-altitude headache are very limited, but adult criteria can be applied in younger patients because they are nonspecific and very general [44]. High-altitude headache may be associated with physiologic changes such as vasogenic edema, pulmonary edema, and hematologic disorders. Therefore, since headache is a nonspecific symptom, especially in young patients, a sentence should be added regarding the exclusion of other factors that may be associated with headache such as ophthalmological disturbances*,* sleep disturbances, and acute cerebral dysfunction of extreme altitude related to additional acute cerebral hypoxia. Headache is also a common symptom of chronic mountain sickness, manifested by physical and mental tiredness, feeling of sadness, shortness of breath upon awakening, palpitations, muscle and/or joint pains, cyanosis of the lips, face, and hands, venous dilation in hands and feet, paresthesias in the distal extremities, dizziness, and tinnitus. Parents should be alert to these symptoms. In acute mountain syndrome, headache may be the only complaint [45–47].

#### Recommendations

Ruling out high-altitude disease is very important, especially in very young individuals in whom verbal communication is poor, as well in all secondary headaches in young patients and as in some adults.

### Headache attributed to airplane travel

#### Comments

In the pediatric age group, headaches caused by air travel in are not necessarily unilateral, and the pain may be of any type. "As with other secondary headache the description of headache by pediatric patient may be not specific" Parents may sometimes infer the headache. If possible, sinus disease should be ruled out. A past history of any type of headache should be considered, because airplane travel may act as a trigger in patients predisposed to headaches [48, 49].

#### Recommendation

We need more data in children and adolescents to make good recommendations.

### Diving headache

#### Comments

The ICHD-3 beta criteria for diving heading have moderate validity but poor reliability. They are more applicable to adolescents than young children. Children are more prone to be diving complications (dizziness, personality changes, confusion, and headache) than adults, and various diving organizations have prohibited diving in children less than 12–15 years old (depending on the organization) [50].

#### Recommendations

1. The criteria for pediatric diving headache should be changed to headache that appears at a depth.
2. Adult criteria may be applied in adolescents more than 15 years old, when diving complications are similar to those in adults.
3. Diving headache may also be due to severe complications of diving to which children less than 12 years old are highly prone. Therefore, a remark should be added that the diagnosis should be made retrospectively after few days, if no other diving complications appear.

### Sleep apnea headache

#### Comments

The criteria for sleep-apnea-related headache have moderate reliability but poor validity in the pediatric age group [51, 52]. Criteria B (apnea-hypopnea index >5) cannot be applied to children and adolescents since different norms of polysomnographic respiratory values are reported for different age groups (infancy, toddlers, adolescents). There are few reports of the characteristics of sleep apnea headache in pediatric patients [52]. As opposed to adults, the most common pathophysiologic factor associated with obstructive sleep apnea in pediatric patients is adenoid hypertrophy in young children and obesity in adolescents [51–53].

#### Recommendations

Criteria B should be accepted, with the addition of the following remark/change: Sleep apnea has been diagnosed according to normal age- related polysomnographic respiratory values. The pathophysiologic factors of adenoid hypertrophy in children and obesity in adolescents should be added to the ICHD-3 criteria.

Key point: Usually, if adenoid hypertrophy or obesity is diagnosed as a cause of sleep apnea, the headache is significantly alleviated or eliminated after adenoidectomy, weight loss, or treatment with positive airway pressure.

### Dialysis headache

#### Comments

The precise mechanism of dialysis headache is not fully understood. Laboratory findings in adults include an increase in levels of nitric oxide, CCRP, and urea, low bicarbonate level, electrolyte imbalance, and hypertension. As these may complicate pediatric dialysis as well, the same diagnostic criteria are probably applicable to both adults and children [54, 55].

#### Recommendation

We need more data in children and adolescents to make good recommendations.

### Headache attributed to arterial hypertension

#### Comments

Hypertension is defined as an average systolic and diastolic blood pressure 20 mmHg above the 95th percentile for age, sex, and height. Pediatric hypertension resembles adult hypertension, although the risk factors differ [56]. The ICHD-3 beta criteria for arterial hypertension-related headache are highly reliable for older children, in whom there is a higher incidence of essential hypertension, but moderately reliable in young children, in whom arterial hypertension is usually secondary to another disease. Children have a high prevalence of chronic renal, vascular, and endocrine background diseases that can cause headache. Concomitant high blood pressure and headache has been reported in the 7–18-year age group; the headache resolves with treatment of the hypertension [57–59].

#### Recommendations

1. Changing the B criteria would increase the generalizability for children and adolescents as follows:

B: Because the definition of childhood hypertension is based on the normative distribution of blood pressure in healthy children, each measurement must be related to blood pressure standards based on age, sex, and height.

2. In the E criteria, an underlying systemic disease as a cause for both hypertension and headache should be excluded.

3. We suggest that a pediatric nephrologist be consulted to ensure that the patient has real hypertension according to the published definitions. White coat hypertension should be excluded as well.

### Headache attributed to pheochromocytoma

#### Comments

There is sacristy of reports in the literature regarding pediatric headache relating to pheochromacytoma [60–64]. There is no specific description for headache was given but in a case report a thunderclap headache was reported. Since no specific description is given to the headache in adult criteria, these criteria may be implied for pediatric patients.

### Headache attributed to hypertensive crisis without hypertensive encephalopathy

#### Comments

The ICHD-3 beta criteria have high reliability but moderate validity, with overlap between adult and pediatric symptoms. Hypertensive crisis is defined as a sudden and abrupt elevation in blood pressure from baseline. The exact values are not fully established in children [65, 66].

#### Recommendations

The criterion of pulsating headache, which is difficult to diagnose in children, should be changed as follows: Pulsating-type headache is not a mandatory finding in children with suspected hypertensive crisis; any type of headache is acceptable as a criterion provided the hypertension and headaches are not better accounted for by an underlying disease.

### Headache attributed to hypertensive encephalopathy

#### Comments

Most studies describe the same symptoms as described in adults criteria, but headache accompanied symptoms that appear in section 3 of the adult criteria are not described in the pediatric literature [65–68].

#### Recommendations

We recommend accepting the adult criteria but section 3 of the accompanied symptoms should not be a criteria.

We suggest section to delete as follows;

3. headache has at least two of the following three characteristics:

- diffuse pain
- pulsating quality
- aggravated by physical activity

### Headache attributed to pre-eclampsia or eclampsia

#### Comments

The ICHD-3 beta criteria for pre-eclampsia/eclampsia-associated headache have high reliability and validity in adolescents since headache symptoms at this age group resemble adult headache. Perpubertal children are not expected physiologically to be pregnant. The symptoms overlap with adult pre-eclampsia or eclampsia. The risk is higher up to age 19 years, so the rate of headache may be higher in that age group as well [69]. Severe vasoconstriction often develops in women with pre-eclampsia, especially when blood pressure is poorly controlled, and can cause brain infarction and hemorrhage. Reversible cerebral vasoconstriction syndrome (RCVS), also referred to as postpartum angiopathy and Call-Fleming syndrome, can develop during the puerperium in the absence of hypertension or other features of pre-eclampsia. Pre-eclampsia, eclampsia, and RCVS can all be complicated by PRES [70].

#### Recommendations

Since headache may be the presenting symptom of other pregnancy- related complications, criteria D should be changed as follows:

D: not better accounted for by another ICHD-3 diagnosis or other pregnancy-related neurologic co-morbidity.

### Headache attributed to autonomic dysreflexia

This type of headache is under recognized in adult patients [71]. Adult criteria based on the that summarized data un adult patients. We had a large report regarding to this type of headache [71]. In the literature no headache was reported in children less than 5 years old [72] and the headache characteristics were not defined in the few cases reported in the adolescents [73].

#### Recommendation

We need more data in children and adolescents to make good recommendations.

### Headache attributed to hypothyroidism

#### Comments

The ICHD-3 beta criteria for hypothyroidism-associated headache have high reliability in adolescents owing to the overlap of clinical symptoms in this age group with adults. Reliability is only moderate for younger patients who may have atypical symptoms. The only report regarding hypotyroidism in pediatric patients described migraine feature of hypothroidsm [74, 75].

#### Recommendation

We need more cases in children and adolescents to make good recommendations.

### Headache attributed to fasting

#### Comments

The types of headache described in the literatures regarding fasting headache is Tension-type headache, migraine and cluster headache [76–78]. The ICHD-3 beta criteria for fasting-related headache have high reliability for adolescents. There are no data for children in the literature, although our experience shows that fasting is a predisposing factor for headaches in this age group. The validity is also high for adolescents, who are included in the published studies, but poor for young children. We presume that in children symptoms may appear sooner after the start of fasting owing to their lower glycogen storage capacity and higher sensitivity to dehydration. Furthermore, most studies were performed in adults after prolonged fasting/starvation (during Ramadan in Muslims or after Yom Kippur in Jews), without any satifying data on childhood headaches [76–78].

#### Recommendation

Headache may appear earlier in the young patients.

### Cardiac Cephalalgia

#### Comments

Cardiac cephalalgia is very rare and data in the literature are limited including children and adolescents [79].

### Headache attributed to disorder of homoeostasis

#### Comments

We need more cases in children and adolescents to make good recommendations.

### Headache or facial pain attributed to disorders of the cranium, neck, eyes, ears, nose, sinuses, teeth, mouth or other facial or cervical structure

This general Criteria is “very general” and can be implied for pediatric population since it connects objective finding of above structure (cranium neck etc) with clinical, laboratory or imaging evidence to the pain that is attributed to the lesion [80].

### Headache attributed to temporomandibular disorder (TMD)

In children and adolescents with headache, it is important to look for signs and symptoms of temporo-mandibular joint dysfunction, which occurs with relatively high frequency in the pediatric population. Symptoms in children include pain in the pre-auricular area, pain during masticatory movements, headaches, restricted masticatory movements, and presence of joint noises but at a lower frequency than in adults. The risk is higher intense than in calm children. Both temporo-mandibular joint dysfunction and its associated headaches can occur in early life and become recurrent in adolescence. They are often related to growth and articular remodeling and disappear with time. For example, chewing noises and altered mandibular function can be attributed to changes in the contour of the temporo-mandibular joint with age. However, it is still unknown if these alterations manifest afterwards as pathological symptoms. The headache pain may be constant or throbbing and may mimic tension type headache. It is often triggered by jaw movement or pressure on the masticatory muscles [81–83]. For ICHD-3 our recommendation is as follows;

1. The criterion for restricted mouth opening should be used according to pediatric published criteria [84]
2. The differential diagnosis of headache due to temporo-mandibular joint dysfunction from tension-type headache should be noted.
3. We recommend to use with the above limitation of adult criteria due to the previous report regarding cause and effect [83–85].

### Headache attributed to psychiatric disorder

There is already a comprehensive statement in ICHD-3 beta: In children and adolescents, primary headache disorders (migraine, episodic tension-type headache and especially chronic tension-type headache) are often comorbid with psychiatric disorder. Sleep disorders, post-traumatic stress disorder, social anxiety disorder (school phobia) attention-deficit/hyperactivity disorder (ADHD), conduct disorder, learning disorder, enuresis, encopresis and tic disorder should be carefully looked for and treated when found, considering their negative burden in disability and prognosis of pediatric headache. This statement is proved by pediatric reports regarding headache and psychiatric disorders [85–90].

For ICHD-3 our recommendation is as follows; children with ADHD, developmental disorders, depressive disorders or anxiety disorders have an increased risk for headache disorders. Some of these cases present clinical pictures of primary headache disorders like migraine or TTH.

### Painful cranial neuropathies and other facial pains

#### Classical trigeminal neuralgia (TGN)

There are very few reports of TGN in the pediatric literature. The symptoms of trigeminal neuralgia in the few s cases described in the literature are the same as adults. We believe it would be judicious to include paroxysmal extreme pain disorders in and around the eyes, occipital and/or the submaxillary or submandibular regions commonly presented as a neuropathy [91].

### Limitations of the paper

In pediatric medicine, the age of the patient and the education of the parents may affect the reliability and validity of the diagnosis. There may also be differences in diagnostic accuracy by language and culture. This paper is based on the data reported in the literature and the personal experience of pediatric headache specialists. We did not use a common database to calculate the validity, sensitivity, or specificity of the ICHD-3 beta criteria. We are planning to organize a prospective language-adapted study supported by clinical assessments of video-taped interviews. Furthermore, some of our comments were restricted by a sparsity of data or absence of knowledge on the applicability of specific aspects/points to the pediatric population.

### Implications of the paper

This is the first detailed evaluation of the diagnostic criteria of headache by headache experts from all over the world. All authors based their comments and recommendations on their personal experience with support from the data in the literature specifically pertaining to the pediatric population. This paper supports the distinction between pediatric and adult headache. We trust that with the accumulation of data, the next version of the ICHD will include specific subsections with separate definitions/criteria of pediatric headache.

## Conclusions

- Children are not simply small adults. They have distinct biopsychosocial attributes that play a clear role in the pathogenesis and presentation of secondary headache disorders, with important differences from adults.
- It is important that physicians be alert to the specific characteristics of pediatric secondary headache disorders for effective diagnosis and management.
- The next version of the ICHD should include specific subtopics of pediatric headaches.
